# Human Synovial Mesenchymal Stem Cells Good Manufacturing Practices for Articular Cartilage Regeneration

**DOI:** 10.1089/ten.tec.2018.0219

**Published:** 2018-12-12

**Authors:** Tiago Lazzaretti Fernandes, Heitor Akio Kimura, Carla Cristina Gomes Pinheiro, Kazunori Shimomura, Norimasa Nakamura, José Ricardo Ferreira, Andreas H. Gomoll, Arnaldo Jose Hernandez, Daniela Franco Bueno

**Affiliations:** ^1^ Sports Medicine Group, Institute of Orthopedics and Traumatology, Hospital das Clínicas HCFMUSP, Faculdade de Medicina, Universidade de São Paulo, São Paulo, Brazil.; ^2^ Instituto de Ensino e Pesquisa, Hospital Sírio-Libanês, São Paulo, Brazil.; ^3^ Department of Orthopaedic Surgery, Osaka University Graduate School of Medicine, Osaka, Japan.; ^4^ Center for Advanced Medical Engineering and Informatics, Osaka University, Osaka, Japan.; ^5^ Department of Materials Science, Post Grad Programme on Materials Science, Military Institute of Engineering (IME), Rio de Janeiro, Brazil.; ^6^ Orthopedic Surgery and Sports Medicine, Hospital for Special Surgery (HSS), New York, New York.

**Keywords:** synovia, tissue engineering, mesenchymal stem cells, hyaline articular cartilage, chondrogenic differentiation, immune modulation

## Abstract

***Background:*** Cartilage restoration is a desperately needed bridge for patients with symptomatic cartilage lesions. Chondral lesion is a pathology with high prevalence, reaching as much as 63% of general population and 36% among athletes. Despite autologous chondrocyte implantation versatility, it still fails to fully reproduce hyaline articular cartilage characteristics. Mesenchymal stem cells (MSCs) may be isolated from various known tissues, including discarded fragments at arthroscopy such as synovial membrane. Choice of harvesting site is motivated by MSCs' abilities to modulate immunologic and inflammatory response through paracrine communication. Synovial MSCs have a greater proliferation and strong chondrogenic potential than bone and adipose MSCs and a less hypertrophic differentiation than bone MSCs. Good manufacturing practice (GMP) laboratory techniques for human clinical trials are still novel. To our knowledge, there are only two clinical trials in humans published since today.

***Purpose:*** Therefore, this work aimed to isolate and characterize synovial MSCs and evaluated their differentiation properties according to GMP standards.

***Materials and Methods:*** One-gram tissue sample from three patients of synovia was harvested at the beginning of arthroscopy surgery. MSCs were isolated, expanded, and characterized by flow cytometry.

***Results:*** It was possible to isolate and expand MSCs cultures from synovia, characterize MSCs by flow cytometry using proper monoclonal antibodies, and differentiate MSCs by coloring technique after chondrogenic, adipogenic, and osteogenic differentiations. Cartilage treatment may benefit from these tissue engineering protocols since arthroscopic procedures are routinely performed for different purposes in a previous stage and a favorable chondronegic differentiation cell lineage may be collected and stored in a less invasive way.

***Conclusion:*** Laboratory protocols established according to presented GMP were able to isolate and characterize MSCs obtained from synovia.

**Impact Statement:**

Articular cartilage restoration is a desperately needed bridge for patients with symptomatic cartilage lesions and it rises as a socioeconomic issue with a considerable economic burden. Synovial mesenchymal stem cells (MSCs) have a greater proliferation rate and strong chondrogenic potential than bone and adipose MSCs and a less hypertrophic differentiation than bone MSCs. To our knowledge, there are only two human clinical trials with good manufacturing practice laboratory techniques for synovial MSCs harvesting and differentiation. Cartilage treatment may benefit from these tissue engineering protocols since arthroscopic procedures are routinely performed for different purposes in a previous stage.

## Introduction

Cartilage restoration is a desperately needed bridge for patients with symptomatic cartilage lesions.^1^ Chondral lesion is a pathology with high prevalence, reaching as much as 63% of general population and 36% among athletes.^2,3^ It rises as an immense socioeconomic issue, and the attempted treatment of these lesions is associated with a considerable economic burden.^4^

Articular cartilage is at high risk of damage during initial trauma, and development of osteoarthritis is estimated to cause important physical limitations and decrease of quality of life.^5,6^

Since there is a lack of vascular system and a limited cellularity in articular cartilage tissue, it presents restrained healing capability.^7,8^ As a consequence, cartilage injuries are often related to pain and joint instability that may diminish or even cease the tissue's functionality.^7,8^

Among cell therapeutics solutions, two main examples are observed: autologous chondrocyte implantation (ACI) and mesenchymal stem cells (MSCs). ACI is a two-step procedure that consists of healthy cartilage harvesting through arthroscopy followed by the expanded cell culture and, in a second step, cartilage defect filling.^9,10^ Despite second and third ACI generations' versatility, those techniques use healthy cartilage tissue, “*in vitro*” related chondrocytes dedifferentiation and still fails to fully reproduce the hyaline characteristics of the original articular cartilage.^7,9,11,12^

MSCs may be isolated from various known tissues, such as bone marrow, adipose tissue, dental pulp, and the synovial membrane.^5,9^ Harvesting site is motivated by their abilities to modulate the organism immunologic and inflammatory response and to stimulate neighboring cell migration, proliferation, and survival through paracrine communication.^13^

It is possible to harvest MSCs from usually discarded fragments at arthroscopy surgery, such as synovial membrane and infrapatellar fat pad.^14^

MSCs from synovia have attracted considerable attention as a promising cell source for chondrogenic tissue engineering.^7^ Synovial MSCs have a greater proliferation and strong chondrogenic potential than bone and adipose MSCs and a less hypertrophic differentiation than bone MSCs.^7,15,16^

Knowledge and technology for synovial MSCs human usage according to good manufacturing practice (GMP) is still novel. It is described in literature that only two clinical trials related to synovial MSCs for articular cartilage repair under ISO9001 certification.^16,17^ Shimomura *et al.*^17^ assessed safety and efficacy of a scaffold-free derived from synovial membrane MSCs for cartilage regeneration and stated that no adverse events were recorded, there was improvement for clinical scores, and histology was similar to hyaline cartilage. Sekiya *et al.*^16^ reported that treatment for articular cartilage lesions with synovial MSCs had succeeded. Magnetic resonance imaging, histology score, and clinical outcome have improved.^16^

Tissue engineering protocols for articular cartilage treatment may benefit from favorable synovial MSCs lineage, since they may be harvested from a less invasive and routinely arthroscopic procedure.^18^ The use of synovial membrane MSCs also reduces the amount of healthy tissue needed as on ACI surgical procedure.^14^ Moreover, GMP conformities for human synovial MSCs processing and products has enormous potential on clinical application and translational research.

Therefore, this work aimed to isolate and characterize synovial MSCs and evaluated their differentiation properties according to GMP standards, and, at least, established some perspectives toward their use in articular cartilage tissue engineering.

## Materials and Methods

This study was approved by the Ethics Committee at the Hospital das Clínicas, University of São Paulo, Medical School (CAPPesq: 1.856.912/61980716.0.0000.0068).

Three patients among 18 and 35 years old (two men and one woman) were included in this study. Patients had the ability to play recreational or competitive sports with Tegner scale^19^ of physical activity equal or superior to 5. Inclusion criteria contained patients who undergone arthroscopic surgery for meniscus or anterior cartilage ligament injuries. Patients with previous history of surgery, infection, inflammatory arthritis, and pregnant women were excluded. All patients signed informed consent terms to participate in this research.

### GMP standards

Laboratory facilities are under Brazilian laws and resolutions (National Sanitary Vigilance Agency–ANVISA—RDC No. 214, February 8, 2018)^20^ that regulate advanced cell therapies. According to regulatory local committee, our laboratory facilities have regulatory inspections, provision of regular reports, appropriate staff training, equipment maintenance, risk and adverse event assessment, fully traceable reagent and processes, compilation of cell lineage, and other quality assessments.^21–23^

Laboratory facilities have recommended infrastructure for clean rooms, including airflow and air particulate control (high efficiency particulate air filter), and antechamber for individual protection paramentation.

Only human cells can be processed at advanced cell therapy laboratory site. Moreover, all reagents from cell isolation to cryopreservation are certified, prion free, and apyrogenic.

All procedures, starting from harvesting, were performed at GMP conditions with maximum degree of decontamination and sterility. Harvesting was executed at surgical operating room after antisepsis and asepsis, with sterile instruments and surgical fields.

Synovial MSCs were harvested at the beginning of arthroscopy surgery through anterolateral portal. One-gram tissue sample from three patients was kept in a 50 mL sterile falcon tube immersed in phosphate-buffered saline (PBS, pH 7.4; Gibco; Invitrogen, Grand Island, NY) according to Shimomura *et al.*^9^ and sent to the Tissue Engineering GMP Laboratory at Hospital Sírio-Libanês. Samples were processed up to 6 h after harvesting to avoid cell death and cross contamination.

To analyze the presence of aerobic and anaerobic bacteria and fungi in culture, the automated microbial detection system BacT/Alert™ 3D was used (BacT/Alert; bioMérieux, Durham, NC) and for Micoplasm detection MycoAlert™ was used (MycoAlert PLUS Mycoplasma Detection Kit; Lonza). Any positive result samples were discharged and new harvesting was recommended.

MSCs have been demonstrated as a well-known lineage differentiation pattern. Our group tested previously genetic stability until passage 18 and no chromosomal abnormalities at the 1st or at the 18th passage was observed.^24,25^

### Cell culture

The tissue was washed twice with PBS plus 4% penicillin/streptomycin. Its digestion was performed using 0.2% Collagenase NB 4G Proved Grade (Serva Electrophoresis, Heidelberg) for 90 min at 37°C. This process was ended by adding 4 mL of Dulbecco's modified Eagle's medium/NutrientMixture F-12 (DMEM/F-12; Gibco; Invitrogen) supplemented with 15% Hyclone™ fetal bovine serum (FBS) U.S. Origin (GE, United States), which is virus- and prion-free, and centrifuged at 1500 rpm for 5 min.

The cell pellet was diluted in DMEM/F-12 and 15% Hyclone FBS U.S. Origin (GE, United States) before plating 10^4^ cells in a 25 cm^2^ cell culture flask and stored at 5% CO_2_ and 37°C.^25^

#### Cell culture expansion

For cell culture expansion, MSCs were kept in basal medium (DMEM/F-12, 15% Hyclone FBS; U.S. Origin; GE, United States), 4% penicillin and streptomycin (Gibco; Invitrogen, Carlsbad, CA), and 4% nonessential amino acids solution (Gibco; Thermo Fisher Scientific, Grand Island) at 5% CO_2_ and 37°C. Medium was replaced three times a week and, as we reached 80% minimum confluence, cells were washed with PBS and collected through TrypLE™ Express (Thermo Fisher Scientific) treatment and replated through 1:2 dilutions. The latter procedure was repeated four to five times until characterization.

Regarding our previous studies,^24^ doubling population was achieved after 24 h and was tested for five consecutive days.

### Characterization by flow cytometry

The strains were characterized by flow cytometry after passage five. One million cells (1 × 10^6^) of each population, individually, were used. The cells were stained with monoclonal antibodies, including CD29‐PE, CD34‐FITC, CD44‐PE, CD45‐PE, CD73‐FITC, CD90‐FITC, CD105‐PE, CD166-PE, CD31-FICT and CD117-PE (BD Biosciences, San Jose, CA). An appropriate isotype‐matched control antibody was used for all analyses. Flow cytometry was performed with a FACSCalibur flow cytometer (BD Biosciences), and the data were analyzed using Cell Quest Software (BD Biosciences).

### Cell differentiation

All three MSCs strains (fourth or fifth passage) were introduced *in vitro* into osteogenic (21 days), chondrogenic (21 days), and adipogenic (18 days) differentiation with respective specific media using StemPro^®^ Osteogenesis Differentiation Kit, StemPro Chondrogenic Differentiation Kit, and StemPro Adipogenic Differentiation Kit media (Gibco; Invitrogen, Grand Island, NY) and a 12-well cell culture plate (Corning^®^ Costar^®^; Sigma‐Aldrich, St. Louis, MO) (5 × 10^3^ cells/well). The three types of media were prepared according to manufacturer data sheets.

#### Adipogenic differentiation

To confirm adipogenic differentiation, after 18 days, the cell strains were fixed with 4% paraformaldehyde (PFA) for 30 min, washed, and stained with a working solution of 0.5% oil red O (Sigma‐Aldrich) for 20 min and then washed with distilled water. Images were obtained using optical microscopy (EVOS™ XL Cell Imaging System; ThermoFisher Scientific).

#### Chondrogenic differentiation

To confirm chondrogenic differentiation, after 21 days, monolayer cells were fixed with 4% PFA for 10 min, and Alcian Blue 8GX (Sigma‐Aldrich) was used to stain the extracellular matrix mucopolysaccharides. The staining solution was prepared by dissolving 1% Alcian Blue 8GX in distilled water containing 3% acetic acid. This solution was filtered and added to each culture well for 2 h; then, the cells were washed with distilled water. Images were obtained using optical microscopy (EVOS XL Cell Imaging System; ThermoFisher Scientific).

#### Osteogenic differentiation

To confirm osteogenic differentiation, after 21 days, cells were fixed with 70% ethanol and incubated in 0.2% Alizarin Red S (Sigma‐Aldrich) for 30 min. Then, they were washed with PBS (Gibco; Invitrogen, Grand Island, NY), and images were obtained using optical microscopy (EVOS XL Cell Imaging System; ThermoFisher Scientific).

## Results

### Cell culture

**FIG. 1.**
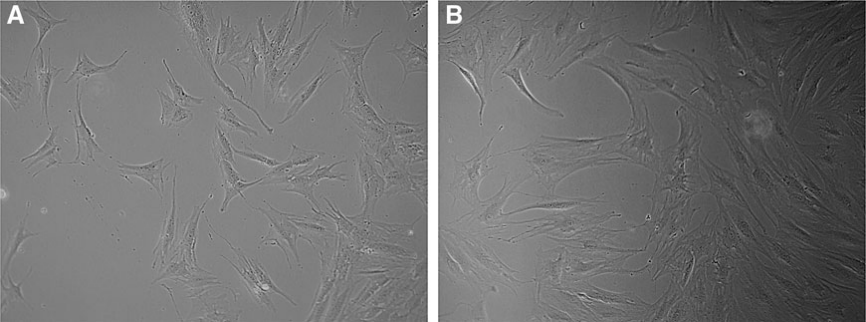
Mesenchymal stem cells at passages four **(A)** and five **(B)**, respectively. Note plastic-adherent characteristic and elongated fibroblastic-like format. Augmentation: 10 × . (Inverted Microscope Olympus CK40).

MSCs were successfully obtained from synovial samples of knee arthroscopy of three patients. Synovial membrane MSCs presented fibroblast-like morphology and plastic adherence, a well-described property of MSCs^26^ (Fig. 1).

### Flow cytometry analysis

**FIG. 2.**
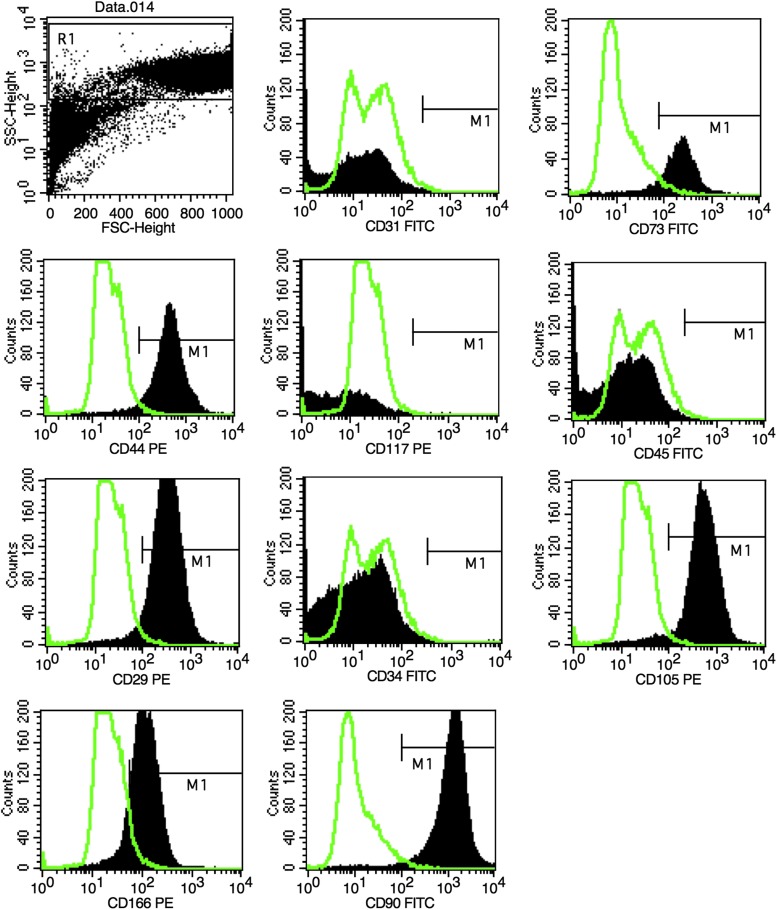
Flow cytometry analysis showing positive reactions to mesenchymal markers (CD29, CD73, CD105, CD90, CD166, and CD44) and negative reactions to hematopoietic (CD34, CD45, and CD117) and endothelial markers (CD31).

All three strains expressed high levels of adhesion markers (CD29 and CD90) and MSC markers (CD44, CD73, CD105, and CD166), and no strains expressed hematopoietic cell markers (CD45, CD34 and CD31), and endothelial cell marker (CD117) as shown in Figure 2.

Example of M1 percentages for each surface marker is shown in Table 1.

**Table 1.** Synovial Membrane Mesenchymal Stem Cells Characterization: M1 Percentages of Positive and Negative Surface Markers

*Positive surface marker*						*Negative*			
*CD29*	*CD44*	*CD73*	*CD90*	*CD105*	*CD166*	*CD34*	*CD45*	*CD31*	*CD117*
93.0%	93.5%	83.3%	97.5%	95.1%	57.1%	4.8%	4.1%	4.8%	5.0%

### Mesenchymal differentiation

**FIG. 3.**
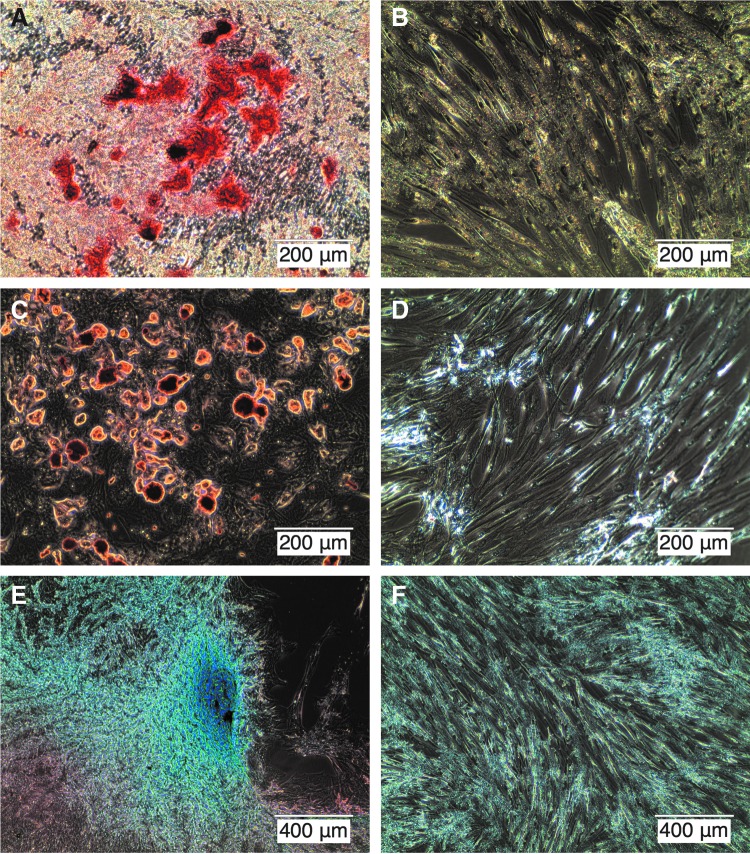
Mesenchymal stem cells osteogenic **(A)**, adipogenic **(C),** and chondrogenic **(E)** differentiations and controls **(B, D, F)**, respectively.

All three MSCs strains were induced to undergo osteogenic, chondrogenic, and adipogenic differentiation, which showed that these strains had mesenchymal origins and maintained multipotentiality (Fig. 3).

## Discussion

Current reports stated that, in comparison with bone marrow-derived stem cells, synovial and infrapatellar fat pad-derived stem cells present improved multipotentiality, great proliferative hate, and higher antiapoptosis potential.^7,27^

Sakaguchi *et al.*^28^ demonstrated superiority of synovial MSCs for clinical application, as its lineage had the greatest ability for chondrogenesis. Progenitor cells from synovia have a predictable pathway for cartilage formation, due to its direct vicinity, they are rich in collagen type II and glucosaminoglycans, and hyaline cartilage observed on metaplasia characterized by synovial chondromatosis.^7,29^

According to International Society for Cell Therapy (ISCT), MSCs are defined as plastic adherent when maintained in standard culture conditions, specific surface antigen expression and the cells must be able to differentiate to osteoblasts, adipocytes, and chondroblasts under standard “*in vitro*” differentiating conditions.^26^ MSC population must express CD105, CD73, and CD90, as measured by flow cytometry and ISCT recommends lack of expression of hematopoietic antigen to be used as additional criteria for MSC.^26^

The described protocols used at Tissue Engineering Laboratory, Hospital Sírio-Libanês for MSCs isolation of synovia as well as the expansion and characterization of these cells showed sufficient effectiveness as it was possible to achieve positive reaction for specific surface antigens CD29, CD73, CD44, CD90, CD105, and CD166 and their differentiation into cartilage, bone, and adipocytes. In addition, according to Figure 1, it was shown that the synovial-derived cells are plastic adherent and exhibit fibroblast-like morphology.

According to some of our previous studies,^24^ other superficial markers were utilized to characterize synovial membrane MSCs. Complementary markers CD117 (important cell surface marker used to identify certain types of hematopoietic progenitors) exhibited hematopoietic lack of expression and CD31 (platelet endothelial cell adhesion molecule—PECAM-1) exhibited endothelial lack of expression. CD73, CD90, and CD105 demonstrated common markers of MSCs. Regular CD34 and CD45 markers evidenced hematopoietic lack of expression. These synovial-derived MSCs also expressed certain adhesion makers (CD29, CD44), which may suggest playing a role in cartilage healing, as well as CD166, which may be utilized to differentiate MSCs from fibroblasts. ISCT guidelines^26^ say, “we encourage investigators to test for as many surface markers (both positive and negative) as they deem important, especially as it relates to their own research.”

High expression (≥95%) of CD73, CD90, and CD105 and the lack of expression (≤2%) of CD34 and CD45 were previously mentioned as ISCT's criteria for human MSCs recognition^26^ and it was also observed in this study. Moreover, we also tested additional specific surface antigens CD29, CD44, and CD166.

CD44, as previously described by Aruffo *et al.*,^30^ settles itself as the main surface receptor for extracellular proteins of hyaluronate, which is exposed in a cartilage lesion. Baboolal *et al.*^31^ also stated that synovial fluid MSCs are capable of adhering to cartilage in a favorable environment. Under cartilage repair and regeneration context, it is believed that hyaluronate plays an important role in repair processes, as it provides a highly hydrated and rather compression-resistant matrix for cell migration.^32,33^ Furthermore, Kikuchi *et al.*^33^ studied the effect of high molecular weight hyaluronan on cartilage degeneration and concluded that there is a direct correlation between hyaluronan molecular weight and its effectiveness on osteoarthritis inhibition. Therefore, CD44 positivity may also be a key condition on the use of MSCs for general cartilage repair and regeneration techniques.

CD29, also known as Integrin Subunit β-1, belongs to the Integrin family of membrane receptors and, therefore, is involved in cell adhesion and recognition in a variety of processes, such as tissue repair.^30^

Lastly among additional surface markers, there is CD166. Halfon *et al.*^34^ reported an interesting observation regarding the distinction between MSCs and fibroblasts; level of expression of CD166 was significantly higher in MSCs than in fibroblasts. In addition, it is important to notice that other cell types have been acknowledged as multipotent, such as dermal fibroblasts.^35–37^ CD166, hence, could be used as a key parameter for MSC and fibroblast discernment, as it was reported that MSCs express significantly higher levels of this surface marker than the other cell types. As it was shown in our study, a substantial portion of the cell population showed high levels of CD166, which could indicate that, despite a significant number of fibroblasts may be also present, it was possible to identify an MSCs population as well.

We proposed a panel related to and optimized for our research with at least four positive markers for MSCs and four negative markers for hematopoietic and endothelial cells. Combined with plastic adherence property and the most uniquely multipontent “*in vitro*” trilineage^26^ differentiation potential from synovial membrane MSCs, we concluded these cells are MSCs.

This experiment was performed with 15% Hyclone FBS U.S. Origin, which is U.S. Department of Agriculture (USDA) approved (GE, United States). This serum is sterile, virus testing panel, prion free, has complete traceability and low antibodies. We preferred to present our results based on the Japanese clinical trial performed by Shimomura *et al.*^17^ that also used FBS (Moregate Biotech) prion- and virus-free. Other options for GMP human serum or serum-free protocols are available. We also did synovial membrane isolation and expansion tests with different types of serum, including StemPro MSC SFM XenoFree (Gibco™; Life technologies™) that is also serum free and with human serum (human male AB plasma, U.S. origin, sterile filtered; Sigma-Aldrich; Merck, Saint Louis).

It is widely accepted that human MSCs exhibit immune-tolerance capacity, paracrine capacity, and the availability of allogeneic MSCs to repair cartilage lesions has been reported in clinical trials^38^ and translational large animal models.^5,39^ The presented evidence of synovial MSCs allows us to investigate the remaining keystones of the tissue engineering triad. Further steps shall include biomaterials and growing factors selection focusing on “*in situ*” cell proliferation and differentiation into articular cartilage on preclinical and phase I/III human clinical trials.

## Conclusion

Laboratory protocols established according to presented GMP were able to isolate and characterize MSCs obtained from synovia.
